# A Review of Self-regulated Learning: Six Models and Four Directions for Research

**DOI:** 10.3389/fpsyg.2017.00422

**Published:** 2017-04-28

**Authors:** Ernesto Panadero

**Affiliations:** Departamento de Psicología Evolutiva y de la Educación, Facultad de Psicología, Universidad Autónoma de MadridMadrid, Spain

**Keywords:** self-regulated learning, self-regulation, metacognition, socially shared regulated learning, shared regulation of learning, motivation regulation, emotion regulation, learning strategies

## Abstract

Self-regulated learning (SRL) includes the cognitive, metacognitive, behavioral, motivational, and emotional/affective aspects of learning. It is, therefore, an extraordinary umbrella under which a considerable number of variables that influence learning (e.g., self-efficacy, volition, cognitive strategies) are studied within a comprehensive and holistic approach. For that reason, SRL has become one of the most important areas of research within educational psychology. In this paper, six models of SRL are analyzed and compared; that is, Zimmerman; Boekaerts; Winne and Hadwin; Pintrich; Efklides; and Hadwin, Järvelä and Miller. First, each model is explored in detail in the following aspects: (a) history and development, (b) description of the model (including the model figures), (c) empirical support, and (d) instruments constructed based on the model. Then, the models are compared in a number of aspects: (a) citations, (b) phases and subprocesses, (c) how they conceptualize (meta)cognition, motivation and emotion, (d) top–down/bottom–up, (e) automaticity, and (f) context. In the discussion, the empirical evidence from the existing SRL meta-analyses is examined and implications for education are extracted. Further, four future lines of research are proposed. The review reaches two main conclusions. First, the SRL models form an integrative and coherent framework from which to conduct research and on which students can be taught to be more strategic and successful. Second, based on the available meta-analytic evidence, there are differential effects of SRL models in light of differences in students’ developmental stages or educational levels. Thus, scholars and teachers need to start applying these differential effects of the SRL models and theories to enhance students’ learning and SRL skills.

## Introduction

Self-regulated learning (SRL) is a core conceptual framework to understand the cognitive, motivational, and emotional aspects of learning. SRL has made a major contribution to educational psychology since the first papers in which scholars began to distinguish between SRL and metacognition (e.g., Zimmerman, 1986; Pintrich et al., 1993a). Since then, publications in the field of SRL theory have increased and expanded in terms of conceptual development, and there are now several models of SRL (Sitzmann and Ely, 2011). In 2001, a theoretical review was published (Puustinen and Pulkkinen, 2001) that included the most relevant models at that time–those articulated by Boekaerts, Borkowski, Pintrich, Winne, and Zimmerman. However, the field has developed significantly since 2001. A first sign of that evolution is that there are now three meta-analyses of the effects of SRL: Dignath and Büttner (2008), Dignath et al. (2008), and Sitzmann and Ely (2011). A second indicator is that there are now new SRL models in the educational psychology field that did not exist in 2001 (e.g., Efklides, 2011; Hadwin et al., 2011, in press). And lastly, a third aspect is that there is a new handbook^1^ (Zimmerman and Schunk, 2011) that presents a variety of established methods to evaluate SRL. Compared to the previous handbook (Boekaerts et al., 2000), the recent handbook has no sections dedicated to presenting new models, being focused on specific aspects of SRL (e.g., basic domains, instructional issues, methodological issues), which shows that the field has evolved and reached a more mature phase.

It is time, then, to reanalyze what is known based on the development of SRL models by comparing them and extracting what are the theoretical and practical implications. Therefore, the aim of this review is to analyze and compare the different SRL models accordingly with the current state of the art and the new empirical data available.

## Methods

### Criteria for Inclusion

Only models with a consolidated theoretical and empirical background were considered for inclusion. The criteria to select a model were that (a) it should be published in JCR journals or SRL handbooks, thus peer-reviewed; (b) it should be written in English; and (c) it should have a minimum number of cites. Models published earlier than 2010 should have at least 500 references. If the model was published after 2010, it should at least have 20 cites per year.

### Selection Process

As a first step, it was analyzed which of the models included in the 2001 review were still actively used. The models by Boekaerts, Winne, and Zimmerman were included as they are widely used and the authors are active SRL scholars who published in the latest handbook (2011). However, the two other models from the 2001 review–Pintrich and Borkowski–needed further consideration. Pintrich was, unfortunately, not able to develop his work further (Limón et al., 2004), but his model and the questionnaire based on it, the Motivated Strategies for Learning Questionnaire (MSLQ) (Pintrich et al., 1993b), are still widely used in research (e.g., Moos and Ringdal, 2012). Borkowski et al.’s (2000) model, which has a strong basis in metacognition, has had less of a presence in the development of the SRL field in recent years, and the main author has transferred his research focus to “exceptionality” (e.g., learning disabilities). Therefore, it was excluded.

The second decision was to consider new models. Two actions were taken. First, a literature search was performed in PsycINFO using the term “self-regulated learning model” from 2001 onward. Second, I asked eight SRL colleagues to identify new models. Five new models were identified. Efklides’ (2011) model explores how emotion and motivation interact with metacognition, and offers a different interpretation of students’ top–down/bottom–up processing in comparison to Boekaerts’, thereby broadening our understanding of SRL. Hadwin et al.’s (2011, in press) model addresses the social aspects of the regulation of learning, which has been an emerging line of research within the SRL field (Panadero and Järvelä, 2015). Additionally, three others were considered: (a) Wolters (2003), which has a strong focus on motivation regulation; (b) Azevedo et al. (2004), which builds upon the work of Winne and colleagues (e.g., Azevedo and Cromley, 2004, p. 525, fourth paragraph) and describes a micro-level analysis of SRL; and (c) Schmitz and Wiese (2006), which takes Zimmerman’s model as a foundation and proposes some tweaks. While these three models are relevant and the scholars have conducted important empirical research on SRL, it was decided not to include them for two reasons. First, Wolters has a strong focus on motivation and does not cover the whole spectrum of SRL components. Second, Azevedo et al. (2004) and Schmitz and Wiese (2006) have considerable similarities with two other models that were already included (Winne and Zimmerman, respectively).

In sum, the models from Zimmerman, Boekaerts, Winne, and Pintrich, will be analyzed with a new lens based on the research of recent years. Additionally, two new models–those of Efklides and of Hadwin, Järvelä, and Miller–will be compared to those more established models. Next, the models will be discussed in chronological order.

## The Self-Regulated Learning Models

### Zimmerman: A Socio-cognitive Perspective of SRL Grounded by Three Models

Zimmerman was one of the first SRL authors (e.g., Zimmerman, 1986). He has developed three different SRL models, being the first one published in 1989 representing what was the first attempt to explain the interactions that influence SRL.

#### History and Development of the Models

Zimmerman (2013) reviewed his career and the development of his work, framing it into the socio-cognitive theory (i.e., individuals acquire knowledge by observing others and social interaction). Zimmerman’s work started from cognitive modeling research in collaboration with Albert Bandura and Ted L. Rosenthal. Later Zimmerman began to explore how individual learners acquire those cognitive models and become experts in different tasks.

**FIGURE 1:**
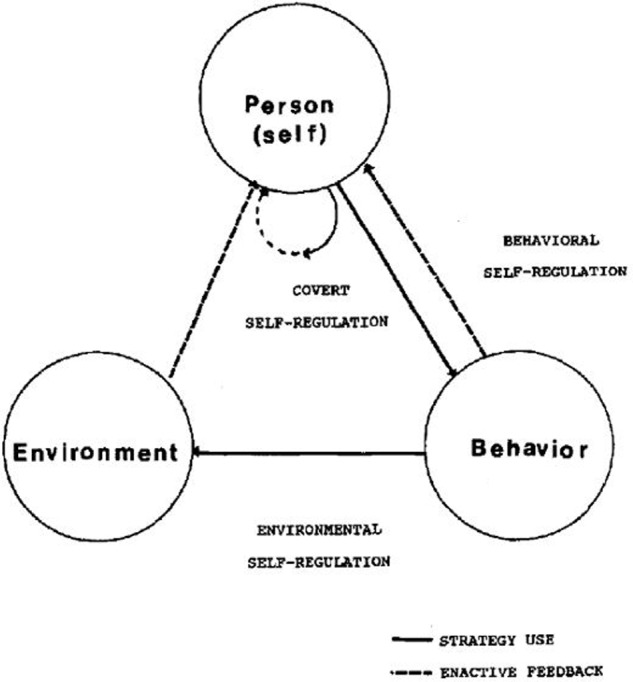
**Triadic model of SRL.** Adapted from Zimmerman (1989).

**FIGURE 2:**
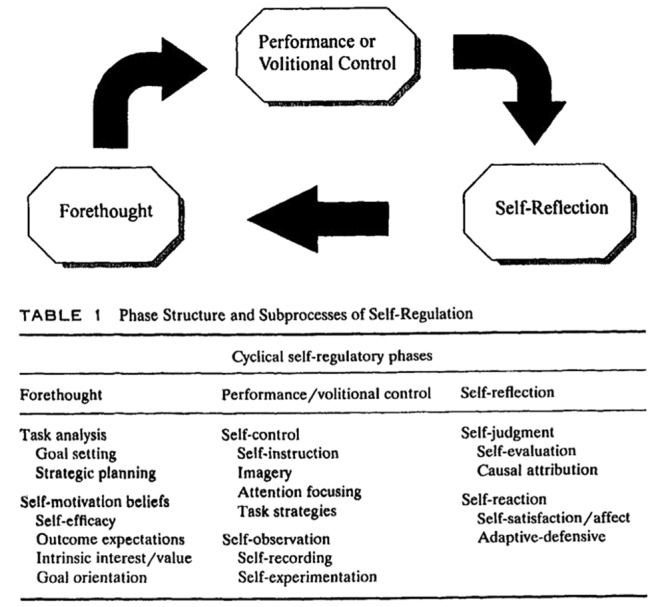
**Cyclical phases model (1st version).** Adapted from Zimmerman (2000).

**FIGURE 3:**
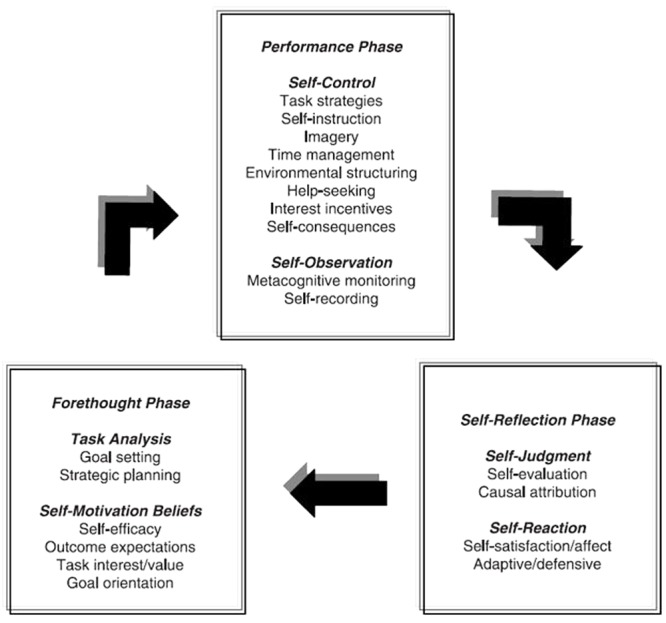
**Current version Cyclical phases model.** Adapted from Zimmerman and Moylan (2009).

**FIGURE 4:**
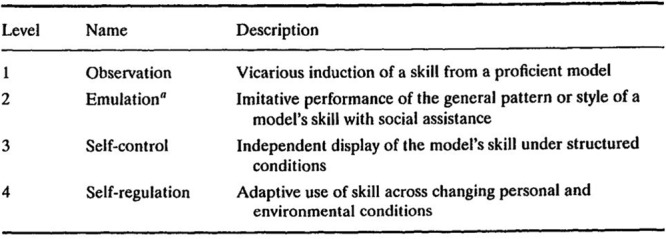
**Multi-level model.** Adapted from Zimmerman (2000).

As one of the most prolific SRL writers, Zimmerman has developed three models of SRL (Panadero and Alonso-Tapia, 2014). The first model (**Figure 1**), known as the Triadic Analysis of SRL, represents the interactions of three forms of SRL: environment, behavior and person level (Zimmerman, 1989). This model describes how SRL could be envisioned within Bandura’s triadic model of social-cognition. The second model (**Figure 2**) represents the Cyclical Phases of SRL, which explains at the individual level the interrelation of metacognitive and motivational processes. This model was presented in a chapter in the 2000 handbook, and it is usually known as Zimmerman’s model. There the subprocesses that belong to each phase were presented, but it was not until 2003 that these subprocesses were embedded in the figure (Zimmerman and Campillo, 2003). Finally, in Zimmerman and Moylan (2009) the model underwent some tweaks (**Figure 3**), including new metacognitive and volitional strategies in the performance phase. The third model Zimmerman developed (**Figure 4**), which recently has been called the Multi-Level model, represents the four stages in which students acquire their self-regulatory competency (Zimmerman, 2000). In this review, Cyclical Phases model will be analyzed, as it describes the SRL process at the same level as the models from the other authors analyzed here.

#### Zimmerman’s Cyclical Phases Model

Zimmerman’s (2000) SRL model is organized in three phases: forethought, performance and self-reflection (see **Figure 3**). In the *forethought* phase, the students analyze the task, set goals, plan how to reach them and a number of motivational beliefs energies the process and influence the activation of learning strategies. In the *performance* phase, the students actually execute the task, while they monitor how they are progressing, and use a number of self-control strategies to keep themselves cognitively engaged and motivated to finish the task. Finally, in the *self-reflection* phase, students assess how they have performed the task, making attributions about their success or failure. These attributions generate self-reactions that can positively or negatively influence how the students approach the task in later performances.

#### Empirical Evidence Supporting Zimmerman’s Cyclical Model

An overview of Zimmerman’s empirical evidence can be found in his career review (Zimmerman, 2013). A special feature of Zimmerman’s empirical research is the use of athletic skills, along with more typical academic skills. A number of studies have been conducted to test different aspects of Zimmerman’s models (Puustinen and Pulkkinen, 2001; Zimmerman, 2013), especially the Multi-level and the Cyclical phase models. Zimmerman conducted work with Kitsantas and Cleary that tested the Multi-level model (Zimmerman and Kitsantas, 1997, 1999, 2002; Kitsantas et al., 2000). Those four studies can be grouped in two types. First, the articles published in 1997 and 1999 studied the differential effect of outcome and process goals with high school students in two different tasks dart throwing and writing, finding support for the model. And second, the articles published in 2000 and 2002 studied the effect of observing different types of models in the development of SRL skills in dart throwing and writing.

The cyclical phase model has been tested in a series of four studies. First, Cleary and Zimmerman (2001) studied the SRL skills showed by adolescent boys who were experts, non-experts and novices in basketball, finding that experts performed more SRL actions. Second, in a similar study, Kitsantas and Zimmerman (2002) compared college women that were experts and non-experts in volleyball, finding that the SRL skills predicted 90% variance in serving skills. Third, Cleary et al. (2006) trained 50 college students in basketball free throws organized in five different conditions: one-phase SRL, two-phases SRL, three-phases SRL, control group practice-only and control group no-practice. The results showed a linear trend: the more phases trained the better the participants’ scores. Finally, fourth, DiBenedetto and Zimmerman (2010) studied 51 high school seniors during science courses seniors finding that higher achievers showed more use of subprocesses from Zimmerman’s model.

Another important piece of research into Zimmerman’s model is the work performed by Bernhard Schmidt and colleagues. As already mentioned, Schmidt has developed a SRL model based on Zimmerman’s and influenced by Kuhl’s (2000) model with changes in the names of the phases and subprocesses included (Schmitz and Wiese, 2006). This theoretical proposal gives a major emphasis to the role of self-monitoring in SRL (Schmitz et al., 2011). Additionally, Schmitz has developed significant research on how the use of learning diaries and its different data analysis known as time-series analysis. His main results have been that the use of learning diaries enhances all SRL phases being an effective way to impact in students’ SRL and performance.

#### Instruments and Measurement Methods

Under Zimmerman’s model umbrella, five instruments and measurements have been developed. First, the subprocesses present in Zimmerman’s model are partly based on the results found in the validation process of the Self-Regulated Learning Interview Schedule (SRLIS) (Zimmerman and Martinez-Pons, 1986, 1988). Second, Zimmerman has developed procedures to assess SRL in experimental training settings for writing and dart throwing (Zimmerman and Kitsantas, 1997, 1999). Third, Cleary and Zimmerman (2001, 2012), Kitsantas and Zimmerman (2002), DiBenedetto and Zimmerman (2010) developed microanalytic measures to assess the validity of the Cyclical Phases model. Fourth, Zimmerman has developed different measures of self-efficacy to self-regulate (Zimmerman and Kitsantas, 2005, 2007) and calibration measures of self-efficacy and self-evaluation (Zimmerman et al., 2011). And, fifth, anchored on the framework of SRL by Zimmerman and Martinez-Pons (1986, 1988), Magno (2010) developed the Academic Self-Regulation Scale (A-SRL) which has been validated analyzing its functional correlation against two well-established SRL instruments the MSLQ and the Learning and Study Strategies Inventory (LASSI) (Magno, 2011).

### Boekaerts: Different Goal Roadmaps (Top–Down/Bottom–Up) and the Role of Emotions

The work by Boekaerts is also one of the earliest in the SRL literature and can be traced back to the late 1980s (e.g., Boekaerts, 1988). Shortly after she presented her first SRL model (Boekaerts, 1991). Her work has focused in explaining the role of goals (e.g., how students activate different types of goals in relation to SRL), and she was the first to use situation-specific measures to evaluate motivation and SRL. In addition, Boekaerts has demonstrated a vast knowledge of the clinical psychology literature on self-regulation and emotion regulation (see Boekaerts, 2011).

#### History and Development of the Models

**FIGURE 5:**
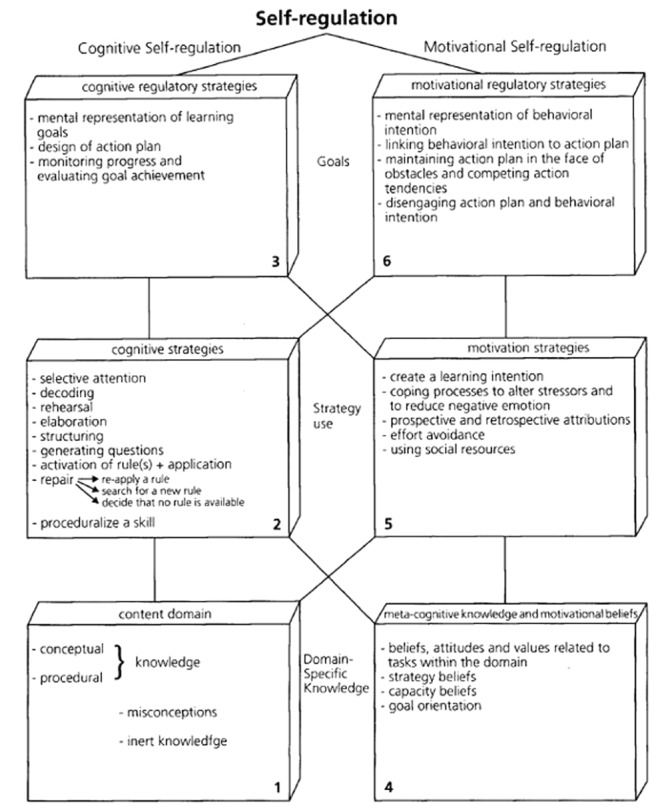
**Six-component model of SRL.** Adapted from Boekaerts (1996b).

Boekaerts has developed two models of SRL. First, she developed a structural model (**Figure 5**) in which self-regulation was divided into six components, which are: (1) domain-specific knowledge and skills, (2) cognitive strategies, (3) cognitive self-regulatory strategies, (4) motivational beliefs and theory of mind, (5) motivation strategies, and (6) motivational self-regulatory strategies (Boekaerts, 1996b). These were organized around, what she then considered to be, the two basic mechanisms of SRL: cognitive and affective/motivational self-regulation. This model has been mainly used to (a) gain more insight into domain-specific components of SRL, to (b) train teachers, to (c) construct new measurement instruments for research, and to (d) design intervention programs (Boekaerts, M. personal communication to author 08/06/2014).

**FIGURE 6:**
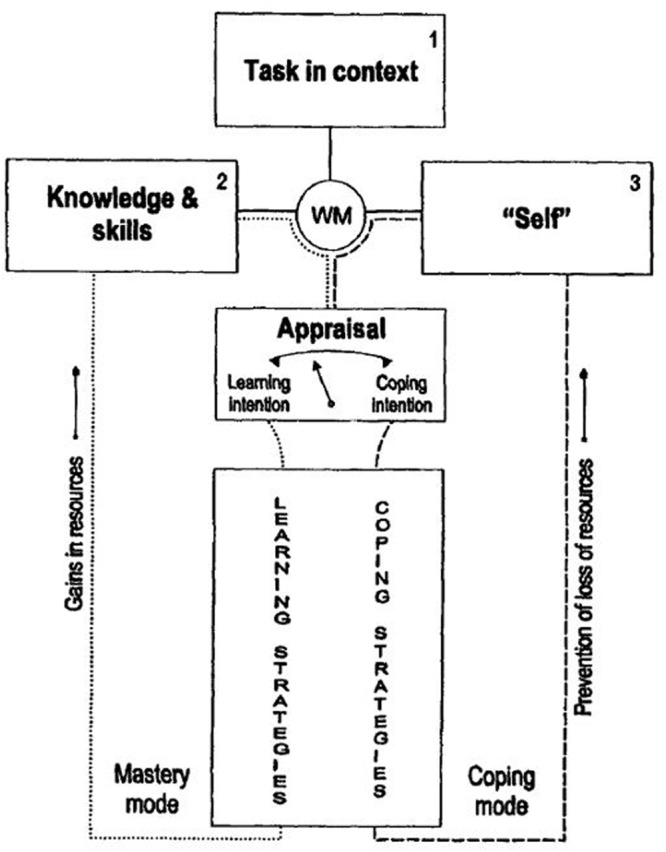
**Model of adaptable learning.** Extracted from the 2000 handbook but cited there as: the original model of adaptable learning. Adapted from Boekaerts (1996a).

**FIGURE 7:**
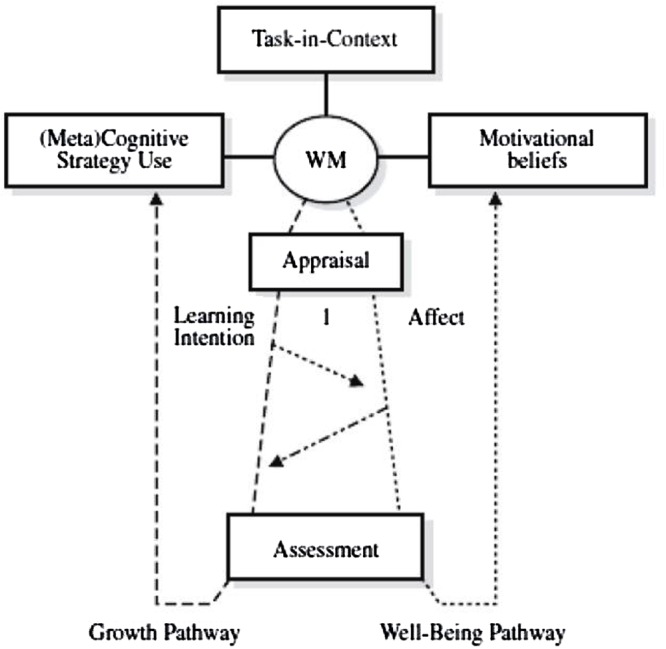
**Dual processing self-regulation model.** Adapted from Boekaerts (2011).

Second, most of Boekaerts’ publications were set up to formulate a second SRL model, namely, the Adaptable Learning Model. This model (see **Figure 6**) was presented at the beginning of the 90s (Boekaerts, 1991, 1992). It describes the dynamic aspects of SRL, and later, evolved into the Dual Processing self-regulation model (**Figure 7**). The Adaptable Learning Model offered a theoretical scaffold for understanding the findings from diverse psychological frameworks, including motivation, emotion, metacognition, self-concept, and learning. The model described two parallel processing modes: (a) a mastery or learning mode and (b) a coping or well-being mode. In a chapter of the 2000 Handbook of self-regulation, Boekaerts and Niemivirta (2000) presented new ideas on goal paths using different figures to visualize how they influence students’ behavior (see pp. 434–435). Although, in 2000, Boekaerts had already presented some notions on her vision of top–down and bottom–up theory, it was not until mid-2000 that these theoretical insights were clearly defined in her model, which was then renamed as the Dual Processing self-regulation model (Boekaerts and Corno, 2005; Boekaerts and Cascallar, 2006). In the 2011 SRL handbook of SR, Boekaerts presented an extended version of this model, which pointed to the different purposes of self-regulation during the learning process, namely, (1) expanding one’s knowledge and skills, (2) protecting one’s commitment to the learning activity, and (3) preventing threat and harm to the self. Boekaerts emphasized the key role that positive and negative emotions play in SRL, and described two different bottom–up strategies, namely, volitional strategies and emotion regulation strategies (**Figure 7**; Boekaerts, 2011).

#### Boekaerts’ Dual Processing Model

In the Dual Processing model (Boekaerts and Cascallar, 2006), the appraisals made by the students are crucial to determine which goal pathway the students will activate. Here, goals are viewed as the “knowledge structures” that guide behavior. For example, if students perceive that the task could be threatening to their well-being, negative cognitions and emotions are triggered. Strategies are then directed to protect the ego from damage, and thereby, students move onto a well-being pathway. On the other hand, if the task is congruent with the students’ goals and needs, they will be interested in amplifying their competence, triggering positive cognitions and emotions, and thereby, moving onto the mastery/growth pathway. Boekaerts (2011) also explains that students who have started a task in the mastery/growth pathway may move to the well-being pathway if they detect cues that they might not be successful.

According to Boekaerts (2011), there are three different purposes for self-regulation:

(a) expanding knowledge and skills…(b) preventing threat to the self and loss of resources so that one’s well-being is kept within reasonable bounds…and (c) protecting one’s commitments by using activities that re-route attention from the well-being pathway to the mastery pathway (pp. 410–411).

The first is what she called “top–down,” as the pursuit of task goals is driven by the students’ values, needs and personal goals (mastery/growth pathway). The second purpose is called “bottom–up,” as the strategies try to prevent the self from being damaged (well-being pathway), and students may experience a mismatch between the task goals and their personal goals. The third purpose occurs when students try to redirect their strategies from the well-being to the mastery/growth pathway, which may happen via external (e.g., teacher or peer pressure) or internal (e.g., self-consequating thoughts) forces. Therefore, emotions are essential in Boekaerts’ model, because when students experience negative emotions, they will activate the well-being pathway and use bottom–up strategies. Pursuant to this interest, Boekaerts has studied, in depth, the different emotion regulation strategies (see Boekaerts, 2011).

#### Empirical Evidence Supporting the Dual Processing Model

Most of the empirical support was provided by Boekaerts and her Ph.D. students using the On-line Motivation Questionnaire (OMQ) – next section for more information- and other specific measures. Their work on the Model of Adaptable Learning concentrated on the top half of the model in **Figure 7**. Four main areas of research can be identified using different measurement tools. First, Seegers and Boekaerts (1993, 1996) studied different aspects of cognitive appraisals and how they determine prospective, anticipatory positive and negative emotions and learning intentions; they found gender differences in the types of appraisals activated. In another publication, Boekaerts (1999) demonstrated that these task specific indices of the students’ interpretations of the learning activity explain more of the variance in learning intention than domain measures, such as self-concept of ability, activation of mastery and performance goals, and interest in the domain.

Second, the effect of prospective cognitions and emotions on learning intention was also studied using the OMQ (Boekaerts et al., 1998; Crombach et al., 2003); a confirmatory factor analysis revealed that seven of the eight presupposed factors could be distinguished empirically, as the internal structure of the tested model was invariant over the academic tasks and also seemed stable over a half-year period.

Third, gender differences in prospective cognitions and emotions were studied using the OMQ and the Confidence and Doubt scale -which measures students’ feelings of confidence every 40 s while they are performing word problems-, (Boekaerts, 1994; Boekaerts et al., 1995; Vermeer et al., 2001). It was found that boys and girls attend differently to math problems, especially word problems. Boys expressed higher confidence, more liking for the tasks, more positive emotions and more willingness to invest effort than girls. Vermeer et al. (2001) using the Confidence and Doubt scale, led to the conclusion that girls view solving math problems basically as applying mathematical rules.

Fourth, several interventions in Dutch secondary vocational schools were conducted that focused on building up metacognitive knowledge and creating opportunities to use deep-level processing (Rozendaal et al., 2003; Boekaerts and Rozendaal, 2006). It was found that the intervention worked best for students who were already familiar with (and used) deep-level processing strategies at the beginning of the study.

Boekaerts has also conducted research on the Dual Processing model and the factors that determine students’ outcome assessments, their reported effort after a task, and their attributions (bottom part of the model). There are two main lines of research here. First, using structural equation models, Boekaerts (2007) looked more closely at the effect of competence and value appraisals on the students’ outcome assessments and reported effort; she also explored the influence that positive and negative emotions during a task have on these outcome variables. She found that students who reported that they had invested effort after doing their mathematics homework, had initially reported that they were competent to do their homework tasks, which produced positive emotions during the task. Valuing a task initially also substantially increased the reported effort. In further research (Boekaerts et al., 2003; Boekaerts, 2007), it was found that outcome assessments after doing homework were positively influenced by both competence and value appraisals. The second line of work, using Neural Network Methodology (family of statistical learning models inspired by the central nervous systems of animals, more specifically biological neural networks) it was examined whether the quality of students’ writing performance (poor/mid/high performance group) could be predicted on the basis of characteristics of the SR system (measured with a specially designed software program based on the OMQ) (Cascallar et al., 2006; Boekaerts and Rozendaal, 2007). It was found that neural networks could predict with high accuracy (ranging 94 and 100%) which students would be in the poor, mid, or high performance groups, based on 56 predictors.

#### Instruments and Measurement Methods

Boekaerts has written a number of reflection papers about the measurement of SRL (the most known Boekaerts and Corno, 2005), and has participated in the creation of four instruments and assessment methods. First, she developed the OMQ (Boekaerts, 1999), which measures the “sensitivity to learn in concrete situations.” It is composed of two parts: (a) students self-report their feelings, thoughts and the effort they want to expend on a concrete task, and (b) after the task, the students report how they feel and their attributions. The validation of her SRL model with the OMQ can be found in Boekaerts (2002). Second, she created an instructional design for secondary vocational schools in the Netherlands based on SRL principles that was called the Interactive Learning Group System (ILGS) innovation (Boekaerts, 1997; Boekaerts and Minnaert, 2003). Third, Boekaerts developed an instrument to record student motivation: the Confidence and Doubt Scale (Vermeer et al., 2001) – explained earlier. And, fourth, she has collaborated with other scholars in the implementation of neural networks for SRL finding high predictive power in such models (e.g., Cascallar et al., 2006).

### Winne and Hadwin: Exploring SRL from a Metacognitive Perspective

Winne and Hadwin’s model of SRL has a strong metacognitive perspective that recognizes self-regulated students as active and managing their own learning via monitoring and the use of, mainly, (meta)cognitive strategies (Winne, 1995, 1996, 1997; Winne and Hadwin, 1998) while asserting the goal driven nature of SRL and the effects of self-regulatory actions on motivation (Winne and Hadwin, 2008). It has been a widely used model, especially in research implementing computer supported learning settings (Panadero et al., 2015b).

#### History and Development of the Model

**FIGURE 8:**
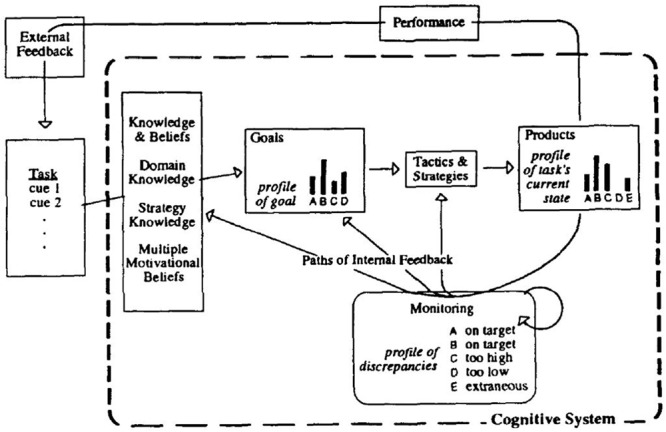
**First version of Winne’s SRL model.** Adapted from Winne (1996).

**FIGURE 9:**
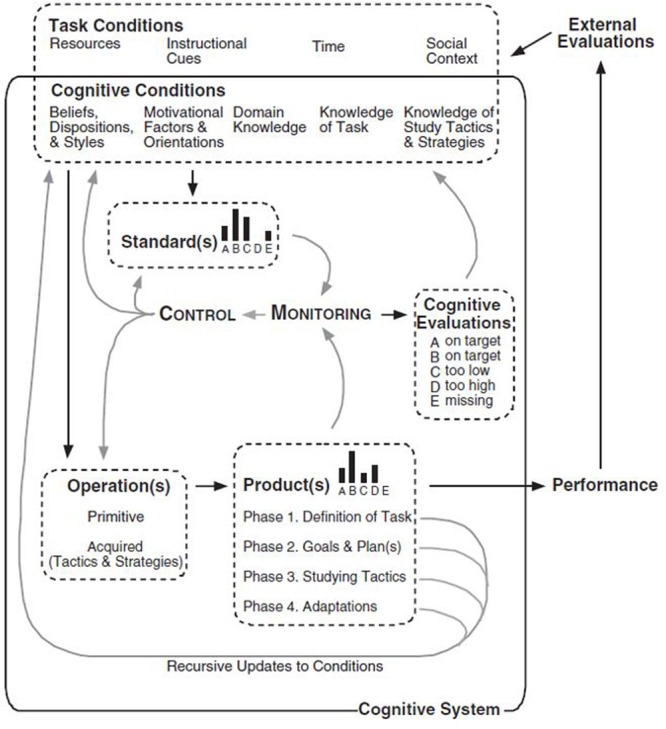
**Current version of Winne’s SRL model.** Adapted from Winne and Hadwin (1998).

Winne and Hadwin’s model is strongly influenced by the Information Processing Theory (Winne, 2001; Greene and Azevedo, 2007), exploring the cognitive and metacognitive aspects of SRL in more detail than the other SRL models with the exception of Efklides’. Some of Phil Winne earliest ideas that led to the model can be traced to his conceptualization of SRL as a fusion of information processing and information processed (Winne, 1995) and Butler and Winne (1995) in their theoretical review of feedback and SRL, in which the concept of internal feedback had a major role and the first version of the model was presented (Figure 1 in Butler and Winne, 1995). Additionally, they presented a second figure in which they explored the different profiles a goal can take and the discrepancy between the goal aims and the current state of work monitoring (Figure 2 in Butler and Winne, 1995). In 1996, Winne presented an updated version of his model (**Figure 8**) in which the two just mentioned figures were fused into one, along with a reflection about the metacognitive aspects that explains the differences in SRL (Winne, 1996). In 1997, he presented the COPES script ideas -see next section- (Winne, 1997). Finally, in 1998, a new version of his model was released (**Figure 9**) including more details and a clearer presentation of COPES (Winne and Hadwin, 1998). It is usually the latter work that is cited when the model is referenced: Winne and Hadwin instead of Winne’s model. That denomination is also used in this review for now onward to keep the consistency with the SRL community, but it is important to keep in mind that the model was firstly presented in previous work (Winne, 1996, 1997). Additionally, these two authors, while collaborating in usual basis, have followed different paths within SRL research as signaled by different chapters in the 2011 SRL handbook (Hadwin et al., 2011; Winne, 2011). Winne has continue examining (meta)cognitive aspects of the model, such as his work on gStudy and nStudy (Winne et al., 2010). Furthermore, he performed minor enhancements to the model although the figure that illustrates the process remains the same (Winne, 2011). Hadwin, while continuing collaborating in the empirical evidence of the model (Winne et al., 2010; Winne and Hadwin, 2013) has additionally focused on the situational, contextual and motivational SRL aspects in collaborative learning settings. This line of work has produced the model of Socially Shared Regulated Learning (SSRL) in collaboration with Järvelä and Miller (see Hadwin, Järvelä, and Miller: SRL in the Context of Collaborative Learning). This present section will explore in more detail the work by Winne as the one by Hadwin will have its own section.

#### Winne and Hadwin’s Model of SRL

According to Winne and Hadwin’s model (e.g., Winne, 2011), studying is powered by SRL across four linked phases that are open and recursive and are comprehended in a feedback loop. These four phases are (**Figure 9**): (a) *task definition*: the students generate an understanding of the task to be performed; (b) *goal setting and planning*: the students generate goals and a plan to achieve them; (c) *enacting study tactics and strategies*: the use of the actions needed to reach those goals; and (d) *metacognitively adapting studying*: occurs once the main processes are completed and the student decides to make long-term changes in her motivations, beliefs and strategies for the future. Winne especially emphasizes that mistakes can be detected in a posterior phase to the one in which they occurred.

Additionally, SRL deploys five different facets of tasks that can take place in the four phases just mentioned (Winne and Hadwin, 1998). These five facets are identified using the COPES acronym, that was used for the first time in Winne (1997) -i.e., Carla COPES with an arithmetic worksheet- (p. 399). It stands for (a) *Conditions*: resources available to a person and the constrains inherent to a task or environment (e.g., context, time); (b) *Operations*: the cognitive processes, tactics and strategies used by the student that are referred to as SMART -Searching, Monitoring, Assembling, Rehearsing and Translating- (Winne, 2001) (e.g., planning how to perform a task); (c) *Products*: the information created by operations (e.g., new knowledge); (d) *Evaluations*: feedback about the fit between products and standards that are either generated internally by the student or provided by external sources (e.g., teacher or peer feedback); and (e) *Standards*: criteria against which products are monitored (definitions taken from Winne and Hadwin, 1998; Greene and Azevedo, 2007) (e.g., assessment criteria).

Furthermore, Winne (2011) model explains in detail how students’ cognitive processing operates while planning, performing and evaluating a task. A crucial aspect is the use of criteria and standards to set goals, monitor and evaluate, aspects which are aligned with self-assessment research (Andrade, 2010; Panadero and Alonso-Tapia, 2013). The model describes how students constantly monitor their activities against standards and use tactics to perform tasks (Winne and Hadwin, 1998). One salient feature is that, in the model figure there is no reference to emotions, and there is only an allusion to motivation. Regardless of this Winne and Hadwin also agrees that SRL is goal-driven in nature and has built connections between his model and research by Pintrich (2003) and Wolters (2003) on regulation of motivation (Winne and Hadwin, 2008).

#### Empirical Evidence Supporting Winne and Hadwin’s SRL Model

Greene and Azevedo (2007) reviewed the empirical evidence for the model. Although they presented it as a theoretical review, due to the fact that they did not perform a “*comprehensive review of the empirical literature*” (p. 338), they reviewed a compelling number of studies (113) that provide empirical support for the model. The review covered all the aspects considered in the model, and made inferences that may have been beyond the initial scope of the work (e.g., they included a section for emotion, which is not explicitly mentioned in the original model). In their conclusions, they stated the model’s potential for future research and pointed out four challenges that needed additional clarification. First, phase four and external evaluations, especially clarifying long-term changes in the students’ SRL and more details on how phase four works (e.g., describing the role of conditions as products of the SRL activity). Second, they made a call for Winne and Hadwin’s model to incorporate the regulation of motivation, using Wolters (2003) as a reference to build the connection. Third, Greene and Azevedo recommended a discussion of how SRL skills develop over the life span. And, fourth, they made a call to consider how student characteristics (e.g., learning disabilities) might impact SRL.

In the later years, Winne and his team have been building a basis for gathering solid empirical evidence on the model based on the work with computers that scaffold students’ SRL while measuring it at the same time (Panadero et al., 2015b). These will be described in the next section. Additionally, Winne has also been exploring the potential of data mining and learning analytics and their application to SRL (e.g., Winne and Baker, 2013).

#### Instruments and Measurement Methods

No classical measurement instruments have been constructed based on Winne and Hadwin’s model, but there are a number of scaffolding tools that measure traces of SRL using the model as theoretical framework (e.g., Winne et al., 2010). They have developed nStudy and gStudy, which are computer-supported learning environments in which the use of SRL is scaffolded while students’ activities are recorded for trace and log data (Winne et al., 2010; Winne and Hadwin, 2013). Additionally, trace data which was brought to SRL research via Winne’s earlier work (Winne, 1982; Winne and Perry, 2000) has opened up new opportunities for the temporal and sequential analysis of SRL which is showing promising new insights for the field (Azevedo et al., 2010; Malmberg et al., 2013). Furthermore, Winne has written important reflection papers on SRL measurement, especially in Winne and Perry (2000) which emphasized the importance of “on-the-fly” or “online” SRL measures and opened up new approaches to the measurement of SRL (Panadero et al., 2015b); and in Winne et al. (2011) which reviews the SRL methods using trace data.

### Pintrich: Grounding the Field and Emphasizing the Role of Motivation in SRL

Pintrich’s work continues to be important in the field as he made a major contribution toward clarifying the SRL conceptual framework (e.g., Pintrich and de Groot, 1990), he conducted crucial empirical work on the relationship of SRL and motivation (Pintrich et al., 1993a), and his questionnaire -MSLQ- (Pintrich et al., 1993b) continues to be widely used (Schunk, 2005; Moos and Ringdal, 2012).

#### History and Development of the Model

Pintrich was one of the first to analyze the relationship between SRL and motivation empirically (Pintrich and de Groot, 1990), theoretically (Pintrich, 2000), and the lack of connections between motivation and cognition (Pintrich et al., 1993a). Further, he later emphasized and clarified the differences between metacognition and self-regulation (Pintrich et al., 2000) and pointed out the areas of SRL that needed further exploration (Pintrich, 1999). In terms of the model itself, there is only one version of it, the one presented in the first handbook of SRL (Pintrich, 2000).

#### Pintrich’s SRL Model

**FIGURE 10:**
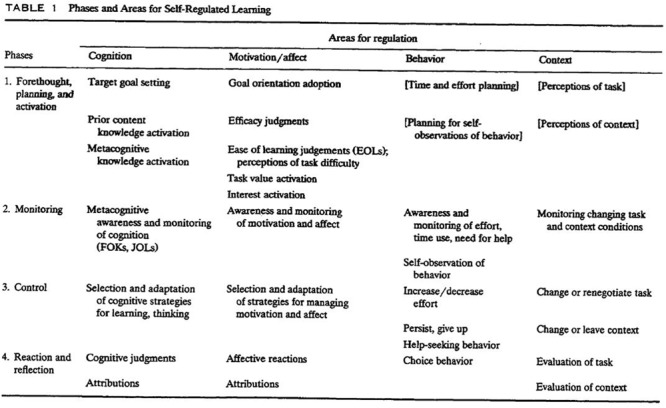
**Pintrich’s SRL model.** Adapted from Pintrich (2000).

According to Pintrich (2000) model, SRL is compounded by four phases: (1) Forethought, planning and activation; (2) Monitoring; (3) Control; and (4) Reaction and reflection. Each of them has four different areas for regulation: cognition, motivation/affect, behavior and context. That combination of phases and areas offers a comprehensive picture that includes a significant number of SRL processes (e.g., prior content knowledge activation, efficacy judgments, self-observations of behavior) (see **Figure 10**). Furthermore, in that chapter, Pintrich (2000) explained in great detail how the different SRL components/areas for regulation are deployed in the different phases. Next how the different areas were conceptualized will be shortly presented. First, in terms of regulation of cognition, Pintrich incorporated metacognitive research such as judgments of learning and feelings of knowing. This incorporation emphasizes how important is cognition for Pintrich’s. Regarding the second area, regulation of motivation and affect, Pintrich explained that motivation and affect could be regulated by the students based on his own empirical work (Pintrich et al., 1993a; Pintrich, 2004). Three years later, Wolters (2003) continued this line of work finding more empirical evidence. The third area, regulation of behavior, is based on the work by Bandura (1977, 1986, 1997) and the Triadic model by Zimmerman (1989). In this area Pintrich incorporated the “*individual’s attempts to control their own overt behavior*” (Pintrich, 2000, p. 466). There is no other SRL model analyzed here that comprehends such area, making Pintrich’s in this sense unique. And, fourth area, the regulation of context which Pintrich included because it addresses those aspects of SRL in which the students attempt to “*monitor, control and regulate the* (learning) *context*” (p. 469).

#### Empirical Evidence Supporting Pintrich’s SRL Model

There is no empirical evidence directly addressing Pintrich’s model validation. However, there is empirical data on the validation of the MSLQ, questionnaire that is the initial empirical work in which Pintrich based his SRL model. That instrument will be analyzed in the next section. Additionally, in a special issue dedicated to his memory, Schunk (2005) reviewed Pintrich’s major contributions to the SRL field identifying six different areas: (a) a conceptual framework and model for SRL (just described in the previous section); (b) the role of motivation in SRL with a special focus on goal orientation; (c) the relationship between SRL, motivation and learning outcomes; (d) the role of classroom contexts in SRL and motivation; (e) the development of SRL through empirical studies; and (f) the development of an instrument to measure SRL (MSLQ).

#### Instruments and Measurement Methods

One major contribution to the SRL field is the MSLQ (Pintrich et al., 1993b). The MSLQ is composed of 15 scales, divided into a motivation section with 31 items, and a learning strategies (SRL) section with 50 items which are subdivided into three general types of scales: cognitive, metacognitive, and resource management (Duncan and McKeachie, 2005). One of the strengths of the MSLQ is its combination of SRL and motivation, which offers detailed information about students’ learning strategies use. Two versions of the questionnaire have been developed for college (Pintrich et al., 1993b) and high school students (Pintrich and de Groot, 1990). For further information on the instrument Duncan and McKeachie (2005) and Moos and Ringdal (2012) provided a list of studies that have used MSLQ. More recently, two reviews have found that the MSLQ is the most used instrument in SRL measurement (Roth et al., 2016) and in self-efficacy measurement (Honicke and Broadbent, 2016). This emphasizes the highly significant impact of Pintrich’s work in SRL.

### Efklides: The Missing Piece between Metacognition and SRL

Efklides (2011) model has a stronger metacognitive background than the other models, except Winne and Hadwin’s which is also metacognitively based. However, when comparing with the latter in Efklides’ model motivation and affect occupy a central role in Efklides’ figure. The model has been cited a significant number of times despite being recently published.

#### History and Development of the Model

Efklides (2011) presented the Metacognitive and Affective Model of Self-Regulated Learning (MASRL) in 2011, which extended her ideas previously published in two theoretical articles (Efklides, 2006, 2008). The model is grounded in classic socio-cognitive theory (Bandura, 1986), as stated by the author herself. Efklides has been influenced by the existing SRL models, along with metacognitive models such as those created by Dunlosky and Metcalfe (2008), Ariel et al. (2009), and Koriat and Nussinson (2009). The distinction of Efklides with the metacognitive models mentioned is that hers is theoretically grounded on previous SRL models (e.g., Zimmerman’s Winne and Hadwin’s, and Pintrich’s). Additionally, Efklides’ model adds to the other SRL models analyzed here, a thorough presentation of the implications of metacognitive models for SRL.

#### MASRL Model

**FIGURE 11:**
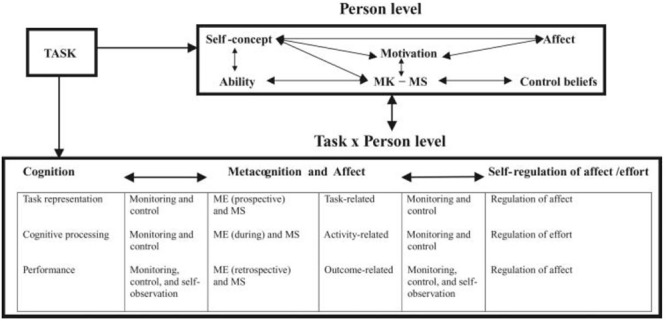
**Metacognitive and affective Model of Self-Regulated Learning model (MASRL).** Adapted from Efklides (2011).

In the MASRL, there are two levels (**Figure 11**). First, there is the Person level-also called macrolevel-which is the most “traditional” view of SRL and comprehends the personal characteristics of the student. In Efklides’ own words: “The Person level represents a generalized level of SRL functioning. It is operative when one views a task resorting mainly on memory knowledge, skills, motivational and metacognitive beliefs, and affect” (Efklides, 2011, p. 10). Therefore, it is composed of: (a) cognition, (b) motivation, (c) self-concept, (d) affect, (e) volition, (f) metacognition in the form of metacognitive knowledge, and (g) metacognition in the form of metacognitive skills. A key aspect is that Efklides considers the Person level to be top–down because it is structured around students’ goals for the task. In other words, the thrust of the student’s goals “guides cognitive processing and the amount of effort” the student will invest, a decision based “on the interactions of the person’s competences, self-concept in the task domain, motivation, and affect, vis-à-vis the perception of the task and its demands” (Efklides, 2011, p. 12).

The second level, the Task × Person level–also known as microlevel–is where the interaction between the type of task and the student’s characteristics –i.e., person level–takes place. This level is bottom–up, as the metacognitive activity takes control of the student’s actions, which causes activity to be “data-driven” with the focus on addressing the demands of the specific task. To put it more simply, the student’s attention moves toward the specific mechanisms of performing the task, and the general learning goal (for example, finishing a summary) is subsumed in a more specific goal (for example, checking for spelling mistakes). Here, the microlevel monitoring is the main process; motivation and affect reactions depend on the evolution of the metacognitive resources and the feedback that comes from the person’s performance – i.e., if s/he is progressing appropriately. Finally, Efklides identifies four basic functions at this level: (a) cognition, (b) metacognition, (c) affect, and (d) regulation of affect and effort, which can be conceptualized independently, vertically, or, in an integrative way, horizontally (see **Figure 11**).

This distinction between the Person level and the Task × Person level is probably the most salient feature of the MARSL model. The Person level represents the general trait-oriented features of students’ SRL, which are goal-driven and top–down. At this level, the MASRL model is similar to other more person-level-oriented models, such as Zimmerman’s (2000). At the Task × Person level, the actions that take place are less conscious and person-oriented: the execution of the task occupies most of the student’s attention and processing, and the actions are data-driven and bottom–up, showing similarities with Winne’s (2011).

In sum, the MASRL model clarifies, in detail, the relationship among metacognition, motivation, and affect via the interaction of the macro and micro levels, and presents a different conceptualization of the top–down/bottom–up implications from the one provided by Boekaerts and Corno (2005). Importantly, the model also illustrates how students perform during the task execution, the phase with the highest cognitive load where all the cognitive resources are leading the activity.

#### Empirical Evidence Supporting the MASRL Model

Efklides (2011) explored the basic MASRL features that have received empirical support by reviewing a compelling amount of evidence from the last two decades. First, she presented the three basic tenets of the model that the empirical evidence needs to address: (a) identifying the MASRL’s two levels (macro- and microlevel), the effects of the task demands on both levels, and what the interactions among them are; (b) the interaction of motivation and affect in the two levels; and (c) the different forms that metacognition takes at both levels. Then she argued that research showing interactions among metacognition, motivation, and affect at the two levels and their interaction actually supports the model. Finally, she presented a large number of studies addressing some of these aspects, grouped in different sections such as “Relations of cognition, metacognition and motivation/affect at the Task × Person level” or “Effects of affect on metacognitive experiences.”

#### Instruments and Measurement Methods

There are two instruments that reflect aspects of the MASRL model. First, Dermitzaki and Efklides (2000) constructed a questionnaire to measure self-concept for a language task. This instrument compares students’ language performance against the four reported categories: self-perception, self-efficacy, self-esteem, and perception of their abilities by others. The interaction of these components is a key aspect of the MASRL model as, for example, these interact at both the Person and the Person × Task levels with metacognition. Secondly, Efklides (2002) created the Metacognitive Experiences Questionnaire, which explores judgments and feelings about cognitive processing. In that paper, the relationship between metacognitive experiences and performance was explored, as well as the effect of task difficulty on metacognitive experiences.

### Hadwin, Järvelä, and Miller: SRL in the Context of Collaborative Learning

Hadwin et al. (2011, in press) and Järvelä and Hadwin (2013), together with other colleagues (for a review, see Panadero and Järvelä, 2015), have explored the potential of SRL theory in explaining regulation in social and interactive features of learning, e.g., use of information and communication technology (ICT) and computer-supported collaborative learning (CSCL) settings. The exploration of SRL and metacognition with this particular purpose is relatively recent, with 2003 identified as the year for the first empirical evidence published (Panadero and Järvelä, 2015). Additionally, the model is strongly influenced by Winne and Hadwin’s (1998) model, as noted in Section “History and Development of the Model.”

#### History and Development of the Model

One of their premises is that, despite the advantages of collaboration and computer-supported collaboration for learning (Dillenbourg et al., 1996), collaboration poses cognitive, motivational, social, and environmental challenges (Järvelä et al., 2013; Koivuniemi et al., 2017). To collaborate effectively, group members need to commit themselves to group work, establish a shared common ground, and negotiate and share their task perceptions, strategies, and goals (Hadwin et al., 2010); in other words, they need to share the regulation of their learning (SSRL). The key issue in SSRL is that it builds on and merges individual and social processes, and it is not reducible to an individual level. It is explained by the activity of the social entity in a learning situation (Greeno and van de Sande, 2007), including situational affordances that provide opportunities for SSRL to happen (Volet et al., 2009).

As mentioned above, SSRL is a field recently developed within SRL. Because of this, the model proposed by Hadwin, Järvelä, and Miller (hereinafter referred to as the SSRL model) has changed significantly from their first proposition in the 2011 handbook to the chapter in the forthcoming SRL handbook.^2^ The two biggest changes incorporated in the latest are: the authors have clarified their perspective on what is Co-regulated Learning (definition below) and they have incorporated and clarified the influence of COPES (Winne, 1997) in their model (Hadwin et al., in press).

#### The Model

**FIGURE 12:**
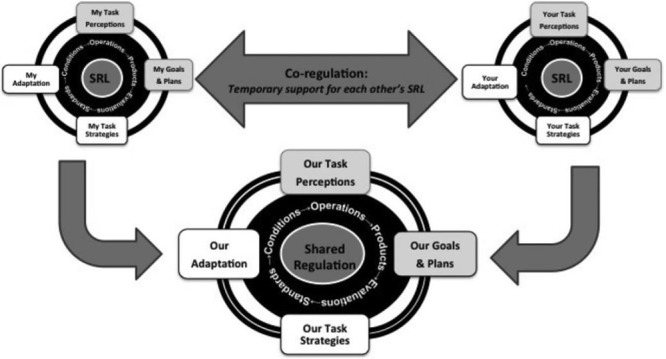
**Socially shared regulated learning model 1.** Adapted from Järvelä and Hadwin (2013).

The SSRL model (Hadwin et al., 2011, in press) proposed the existence of three modes of regulation in collaborative settings: self-regulation (SRL), co-regulation (CoRL), and shared regulation (SSRL) (**Figure 12**). First, SRL in collaboration refers to the individual learner’s regulatory actions (cognitive, metacognitive, motivational, emotional, and behavioral) that involve adapting to the interaction with the other group members.

Secondly, CoRL in collaboration “refers broadly to affordances and constraints stimulating the (student’s) appropriation of strategic planning, enactment, reflection, and adaptation (occurring when in interaction with other students or group members)” (Hadwin et al., in press, p. 5). This regulatory level is the one that has been in the most dispute in the field, as its use has not been consistent (Panadero and Järvelä, 2015).

Finally, the third type, SSRL in collaboration, occurs when “deliberate, strategic and transactive planning, task enactment, reflection and adaptation” are taken within a group (Hadwin et al., in press, p. 5). The key difference between SSRL and CoRL is that, in the former, the regulatory actions “emerge through a series of transactive exchanges amongst group members” whilst in CoRL they are guided or directed by (a) particular group member/s.

What are the significant changes between the 2011 model version and the forthcoming version? First, the CoRL mode has been reconceptualized based on the empirical evidence (Panadero and Järvelä, 2015). Hadwin et al. (2011) proposed three types of CoRL: (a) temporary mediation (by other than the learner) of regulated learning to promote SRL, (b) distributed regulation of each other’s learning in a collaborative task, and (c) a microanalytic approach focusing on interactions through which social environments co-regulate learning. In their forthcoming proposal, they have shifted the focus to the effects of collaborating alone and have not discussed the microanalytic approach in such detail. Another crucial change is that they have considered the reviewed empirical evidence that CoRL and SSRL could both occur “as groups progress through different phases on their collaboration and not always SSRL nor will co-regulation happen in isolation” (Panadero and Järvelä, 2015, p. 199).

**FIGURE 13:**
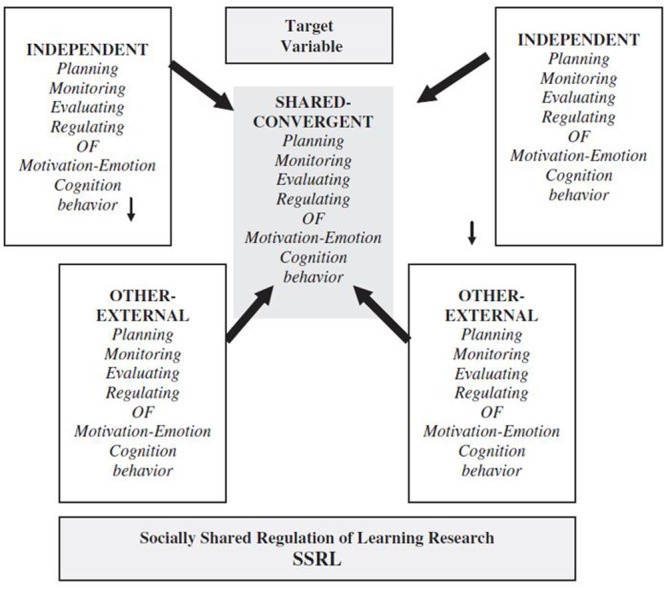
**Socially shared regulated learning model 2.** Adapted from Hadwin et al. (2011).

Hadwin et al. (2011) conceptualized SSRL as unfolding in four loosely sequenced and recursively linked feedback loops (**Figure 13**) taken from Winne and Hadwin (1998). During the first loop, groups negotiate and construct shared task perceptions based on internal and external task conditions. Through the second loop, groups set shared goals for the task and make plans about how to approach the task together. In the third loop, groups strategically coordinate their collaboration and monitor their progress. Based on this monitoring activity, the groups can change their task perceptions, goals, plans, or strategies in order to optimize their collective activity. Finally, in the fourth loop, groups evaluate and regulate for future performance. In essence, when groups engage in SSRL, they extend regulatory activity from the “I” or “you” level to regulate their collective activity in agreement (Hadwin et al., 2011).

In the forthcoming model proposal, the four-phase cycle remains, but under different labels, now using the ones proposed in Winne and Hadwin’s work. Additionally, there is a crucial change: Winne’s (1997) COPES architecture is introduced for the first time in the SSRL model. This addition clarifies, especially, the (meta)cognitive processing at the three regulatory modes along with the effects on motivation and emotion (Hadwin et al., in press, **Figure 1**).

#### Empirical Evidence Supporting the Model

The SSRL authors have been working toward empirical verification of their model (e.g., Järvelä et al., 2013). Meanwhile, other researchers have also conducted a growing number of studies on SSRL. A review on CoRL and SSRL by Panadero and Järvelä (2015) extracted three main conclusions. First, different levels of social regulation were identified: a less balanced type called co-regulation, in which one member of the group takes the lead; and a jointly regulated type, in which goals are negotiated and strategies are shared, known as SSRL. Because of this, those authors proposed to reconceptualize how the CoRL and SSRL modes intertwine, which constitutes one of the main changes in the forthcoming version of the model. Secondly, empirical evidence of the occurrence of SSRL in cognitive, metacognitive, motivational, and emotional shared areas were found. This finding is important, as it shows that shared regulation happens within all SRL areas. And third, there was evidence that SSRL might promote learning and performance. Additionally, new research published after the review has continued strengthening the empirical evidence around the model (e.g., Järvelä et al., 2016a,b).

#### Instruments

At this time, no classical measurement instruments (e.g., questionnaires) have been developed under the SSRL model, even though there is research in the field using self-reported data (e.g., Panadero et al., 2015a). Because of the contextual nature of interpersonal regulation of learning (Vauras and Volet, 2013), new methodologies have been developed to investigate SSRL. Current instruments combine scaffolding and measures in context as, for example, a computer-supported environment to promote group awareness, planning, and evaluation (e.g., Järvelä et al., 2015). Additionally, the joint effort of the model authors has been in developing multi-modal data collections including objective data (e.g., eye tracking, physiological responses) triangulated with subjective data, such as students’ conceptions and intent (Hadwin et al., in press).

## Comparing Self-Regulated Learning Models

Next, the models will be compared in the following categories. First, the models’ number of cites. Second, all the models are divided into different SRL phases and subprocesses. They are compared here, to extract conclusions. Thirdly, there are three main areas that SRL explores; (meta)cognition, motivation, and emotion; therefore, their positioning in each of the six models is analyzed. And, fourth, the SRL models present significant differences in three major aspects of conceptualization: top–down/bottom–up, automaticity, and context.

### Citations and Importance in the Field

One word of advice before starting this section: the number of citations garnered is an indicator that can be influenced by aspects not related exclusively to the quality of the model. Important innovations can actually be made by models that have not received so many cites. Nevertheless, it is an interesting indicator to extract some conclusions from.

In **Table 1**, the number of citations per model is presented. The Efklides and SSRL models have a lower total number, as they were published recently. Nevertheless, they show promising numbers in citations per year, which indicates their relevance. The models of Boekaerts and of Winne and Hadwin’s models form a second group according to their number of citations. It is important to point out that Boekaerts and Corno’s (2005) study includes not only Boekaerts’ model, but also information about Corno’s and Kuhl’s models and, especially, a reflection on SRL measurement. Therefore, it is only a partial representation of citations of Boekaerts’ model, but it is her most cited paper where her model is presented. Winne and Hadwin’s (1998) book is the most cited work regarding their model, but it is not the original presentation of their work, as earlier discussed. Finally, Pintrich’s and Zimmerman’s models, both presented in the 2000 handbook, have the highest number of citations, with Zimmerman as the most cited.

**Table 1:** Number of citations of the different SRL models main publication.

Model	Publication	Total citations	Citations year^∗^
Boekaerts	Boekaerts and Corno, 2005	1011	84.25
Efklides	Efklides, 2011	251	41.83
Hadwin et al.	Hadwin et al., 2011	196	32.67
Pintrich	Pintrich, 2000	3416	200.94
Winne and Hadwin	Winne and Hadwin, 1998	1037	54.58
Zimmerman	Zimmerman, 2000	4169	245.24

*Data as in 20th of March 2017. Search performed via Google Scholar. ^∗^The average citation per year was calculated dividing the total number of citation by the resulting number of subtracting to 2017 -the current year- the year of publication of the reference.*

If we compare the number of the four older models, Pintrich’s and Zimmerman’s models have been more widely used in comparison to Boekaerts’ and that of Winne and Hadwin. There are two probable causes. One is that the first ones are more comprehensive and easier to understand and apply in classrooms (Dignath et al., 2008). With regards to the first cause, both Pintrich’s and Zimmerman’s models include a more complete vision of different types of subprocesses. If we compare these four models figures, it is salient that Zimmerman and Pintrich (a) present more specific subprocesses than Boekaerts and (b) include motivational and emotional aspects that are not directly presented by Winne and Hadwin. The second cause is that Boekaerts’ model and Winne and Hadwin’s are slightly less intuitive, and a deeper understanding of the underpinning theory is needed for a correct application. This is not to say that these two models are less relevant than the others; on the contrary, both cover in depth two critical aspects for SRL: emotion regulation and metacognition. To finalize, Moos and Ringdal’s (2012) review of the teacher’s role in SRL in the classroom found that Zimmerman’s model has been the predominant in that line of research, as it offers “a robust explanatory lens” which might help the most when working with teachers as proposed by these authors.

### Phases and Subprocesses

All of the model authors agree that SRL is cyclical, composed of different phases and subprocesses. However, the models present different phases and subprocesses, and by identifying them we can extract some conclusions. In general terms, Puustinen and Pulkkinen’s (2001) review concluded that the models they analyzed had three identifiable phases: (a) preparatory, which includes task analysis, planning, activation of goals, and setting goals; (b) performance, in which the actual task is done while monitoring and controlling the progress of performance; and (c) appraisal, in which the student reflects, regulates, and adapts for future performances. What is the conceptualization of SRL phases in the two added models? (see **Table 2**). First, Efklides (2011) does not clearly state an appraisal phase in her model, although she considers that the Person level is influenced after repeated performances of a task. Second, the SSRL model in its version from 2011, although strongly influenced by Winne and Hadwin’s, presents four phases that are similar to Pintrich’s but using different labels. Therefore, the SSRL model classification in the table is the same one that Puustinen and Pulkkinen (2001) proposed for Pintrich’s.

**Table 2:** Models’ phases.

Models	SRL phases		
	Preparatory phase	Performance phase	Appraisal phase
Boekaerts	Identification, interpretation, primary and secondary appraisal, goal setting	Goal striving	Performance feedback
Efklides	Task representation	Cognitive processing, performance	
Hadwin et al., 2011	Planning	Monitoring, control	Regulating
*Hadwin et al. (in press)^∗^*	*Negotiating and awareness of the task*	*Strategic task engagement*	*Adaptation*
Pintrich	Forethought, planning, activation	Monitoring, control	Reaction and reflection
Winne and Hadwin	Task definition, goal setting and planning	Applying tactics and strategies	Adapting metacognition
Zimmerman	Forethought (task analysis, self-motivation)	Performance (self-control self-observation)	Self-reflection (self-judgment, self-reaction)

*^∗^The early draft provided by the authors did not provide the exact names for the phases but it could be implied the phases are similar to Winne and Hadwin’s. Therefore, this review comparison will be based on their 2011 publication.*

What can be concluded? Even if all of the models considered here except Efklides’, can be conceptualized around those three phases proposed by Puustinen and Pulkkinen, two conceptualizations of the SRL phases can be distinguished. First, some models emphasize a clearer distinction among the phases and the subprocesses that occur in each of them. Zimmerman’s and Pintrich’s models belong to this group, each having very distinct features for each phase. Those in the second group-the Winne and Hadwin, Boekaerts, Efklides, and SSRL (in its forthcoming version) models-transmit more explicitly that SRL is an “open” process, with recursive phases, and not as delimited as in the first group. For example, Winne and Hadwin’s figure does not make a clear distinction between the phases and the processes that belong to each: SRL is presented as a feedback loop that evolves over time. It is only through the text accompanying the figure that Winne and Hadwin (1998) clarified that they were proposing four phases.

One implication from this distinctive difference could be in how to intervene according to the different models. The first group of models might allow for more specific interventions because the measurement of the effects might be more feasible. For example, if a teacher recognizes that one of her students has a motivation problem while performing a task, applying some of the subprocesses presented by Zimmerman at that particular phase (e.g., self-consequences) might have a positive outcome. On the other hand, the second group of models might suggest more holistic interventions, as they perceive the SRL as a more continuous process composed of more inertially related subprocesses. This hypothesis, though, would need to be explored in the future.

### (Meta)cognition, Motivation, and Emotion

Next, the three main areas of SRL activity and how each model conceptualizes them will be explored. The interpretation is guided by the models’ figures as they reveal the most important SRL aspects for each author. A classification based on different levels for the three aforementioned areas is proposed (**Table 3**). It is important to clarify that the levels were conceptualized, not as being close in nature, but rather, as being positions on a continuum.

**Table 3:** Models figures comparison on cognition, motivation, and emotion.

Levels of relevance	Cognition	Motivation	Emotion
First (more emphasis)	Winne Efklides SSRL	Zimmerman Boekaerts Pintrich	Boekaerts
Second	Pintrich Zimmerman	SSRL Efklides Winne	Zimmerman/Pintrich SSRL
Third (less emphasis)	Boekaerts		Efklides Winne

*Note from the author: these levels should be read as a continuum.*

#### (Meta)cognition

Three levels are considered with regard to (meta)cognition. The first level includes models with a strong emphasis on (meta)cognition. The first model at this level is Winne and Hadwin’s, in which the predominant processes are metacognitive: “Metacognitive monitoring is the gateway to self-regulating one’s learning” (Winne and Perry, 2000, p. 540). Efklides’ model includes motivational and affective aspects, but the metacognitive ones are defined in more detail at the Task × Person level and are the ones with more substance. Finally, the SSRL model includes in the forthcoming version the COPES architecture from Winne and Hadwin. However, due to the fact that the SSRL 2011 version did not emphasize (meta)cognition, it was decided to locate it after the two more metacognitive models. At the second level are Pintrich’s and Zimmerman’s models. Pintrich (2000) incorporates the “regulation of cognition,” which has a central role along with aspects of metacognitive theory such as FOKs and FOLs. Zimmerman (2000) presents a number of leading cognitive/metacognitive strategies, but they are not emphasized over the motivational ones, as is the case for the models just discussed. At the third level, Boekaerts includes the use of (meta)cognitive strategies in her figures, but does not explicitly refer to specific strategies.

#### Motivation

A two-level classification is proposed. The Zimmerman, Boekaerts, and Pintrich models are at the first level. Zimmerman’s own definition of SRL explicitly states the importance of goals and presents SRL as a goal-driven activity. In his model, in the forethought phase, self-motivation beliefs are a crucial component; the performance phase was originally described (Zimmerman, 2000) as performance/volitional control, which indicates how important volition is; and at the self-reflection phase, self-reactions affect the motivation to perform the task in the future. According to Boekaerts, the students “interpret” the learning task and context, and then activate two different goal paths. Those pathways are the ones that lead the regulatory actions that the students do (or do not) activate (e.g., Boekaerts and Niemivirta, 2000). In addition, Boekaerts also included motivational beliefs in her models as a key aspect of SRL (see **Figure 7**). Finally, Pintrich (2000) also included a motivation/affect area in his model that considers aspects similar to those in Zimmerman’s, but Pintrich’s places a greater emphasis on metacognition. It is also important to mention that Pintrich conducted the first research that explored the role of goal orientation in SRL (Pintrich and de Groot, 1990).

The second level includes the SSRL, Efklides, and Winne and Hadwin models. SSRL included motivation in the 2011 version figure and emphasized its role in collaborative learning situations, but without differentiating motivational components in detail. Nevertheless, the authors have conducted a significant amount of research regarding motivation and its regulation at the group level (e.g., Järvelä et al., 2013). Finally, Winne and Hadwin (1998) and Efklides (2011) included motivation in their models, but it is not their main focus of analysis.

#### Emotion

Three levels are proposed. In the first one (Boekaerts, 1991; Boekaerts and Niemivirta, 2000) emphasizes the influence of emotions in students’ goals and how this activates two possible pathways and different strategies. For Boekaerts, ego protection plays a crucial role in the well-being pathway, and for that reason it is essential for students to have strategies to regulate their emotions, so that they will instead activate the learning pathway. At the second level, Pintrich (2000) and Zimmerman (2000) shared similar interpretations of emotions. They both put the most emphasis on the reactions (i.e., attributions and affective reactions) that occur when students self-evaluate their work during the last SRL phase. In addition, both mentioned strategies to control and monitor emotions during performance: Pintrich discusses “awareness and monitoring” and “selection and adaptation of strategies to manage” (Pintrich, 2000), and Zimmerman stated that imagery and self-consequences can be used by students to self-induce positive emotions (Zimmerman and Moylan, 2009). Nevertheless, in the preparatory phases, neither of them mentions emotions directly. Yet, Zimmerman argues that self-efficacy, which is included in his forethought phase, is a better predictor of performance at that phase than emotions or emotion regulation (Zimmerman, B. J. personal communication with the author, 28/02/2014). The SSRL model includes emotion in its 2011 version figure (Hadwin et al., 2011), but the subprocesses that underlie the regulation of emotion are not specified. Nonetheless, these authors clearly argue that collaborative learning situations present significant emotional challenges, and they have conducted empirical studies exploring this matter (e.g., Järvenoja and Järvelä, 2009; Koivuniemi et al., 2017). Finally, Efklides (2011) and Winne and Hadwin (e.g., Winne, 2011) mention the role of emotions in SRL [e.g., “affect may directly impact metacognitive experiences as in the case of the mood” (Efklides, 2011, p. 19, and she included it in her model at two levels]. However, they do not place a major emphasis on emotion-regulation strategies.

### Three Additional Areas for a Comparison

As mentioned earlier, three additional areas in which the models present salient differences were identified.

#### Top–Down/Bottom–Up (TD/BU)

The first model that included this categorization of self-regulation was Boekaerts and Niemivirta (2000). Top–down is the mastery/growth pathway in which the learning/task goals are more relevant for the student. On the other hand, bottom–up is the well-being pathway in which students activate goals to protect their self-concept (i.e., self-esteem) from being damaged, also known as ego protection. Efklides (2011) also uses this categorization, but with different implications. For her, top–down regulation occurs when goals are set in accordance with the person’s characteristics (e.g., cognitive ability, self-concept, attitudes, emotions, etc.), and self-regulation is guided based on those personal goals. Bottom–up occurs when the regulation is data-driven, i.e., when the specifics of performing the task (e.g., the monitoring of task progress) direct and regulate the student’s actions. In other words, the cognitive processes are the main focus when the student is trying to perform a task.

The other models do not explore this categorization explicitly, although some implicit interpretations can be extracted. This way, there could be a third vision of TD/BU that is based on the interactive nature of Zimmerman’s model and Winne and Hadwin’s. Zimmerman (personal communication to author 27/02/2014) explained:

*Historically, top–down theories have been cognitive and have emphasized personal beliefs and mental processes as primary (e.g., Information Processing theories). By contrast, bottom–up theories have been behavioral and have emphasized actions and environments as primary (e.g., Behavior Modification theories). When Bandura (1977) developed social cognitive theory, he concluded that both positions were half correct: both were important. His theory integrates both viewpoints using a triadic depiction. I contend that his formulation is neither top–down [n]or bottom–up but rather interactionist where cognitive processes bi-directionally cause and are caused by behavior and environment. My cyclical model of SRL elaborates these triadic components and describes their interaction in repeated terms of cycles of feedback. Thus, any variable in this model (e.g., a student’s self-efficacy beliefs) is subject to change during the next feedback cycle…. There are countless examples of people without goals who experience success in sport, music, art, or academia and subsequently develop strong goals in the process. Interactionist theories emphasize developing one’s goals as much as following them*.

Winne (personal communication to the author 27/02/2014) stated:

*I didn’t introduce this terminology because it is limiting. A vital characteristic of SRL is cycles of information flow rather than one-directional flow of information. Some cycles are internal to the person and others cross the boundary between person and environment*.

In sum, Zimmerman and Winne do not consider TD/BU to be applicable to their models, as the recursive cycles of feedback during performance generate self-regulation and changes in the specificity of the goals.

As Pintrich’s (2000) model is goal-driven, it could be assumed that it conceptualizes top–down motivation as coming from personal characteristics, as proposed by Efklides (2011). Nevertheless, Pintrich also included goal orientation, which implicates performance and avoidance goals, which has a connection to Boekaerts’ well-being pathway, especially avoidance goals. Therefore, it is difficult to discern with any precision what the interpretation of TD/BU would be for his model. The SSRL model (Hadwin et al., 2011) has not yet clarified this issue, though a stance similar to that of Winne and Hadwin could be presupposed.

#### Automaticity

In SRL, automaticity usually refers to underlying processes that have become an automatic response pattern (Bargh and Barndollar, 1996; Moors and De Houwer, 2006; Winne, 2011). It is frequently used to refer to (meta)cognitive processes: some authors maintain that, for SRL to occur, some processes must become automatic so that the student can have less cognitive load and can then activate strategies (e.g., Zimmerman and Kitsantas, 2005; Winne, 2011). However, it can also refer to motivational and emotional processes that occur without student’s awareness (e.g., Boekaerts, 2011). Next, some quotations from the models on this topic will be presented to illustrate the different perspectives of automaticity. Winne (2011) stated:

Most cognition is carried out without learners needing either to deliberate about doing it or to control fine-grained details of how it unfolds…Some researchers describe such cognition as “unconscious” but I prefer the label implicit…Because so much of cognitive activity is implicit, learners are infrequently aware of their cognition. There are two qualifications. First, cognition can change from implicit to explicit when errors and obstacles arise. But, second, unless learners trace cognitive products as tangible representations -“notes to self” or underlines that signal discriminations about key ideas, for example-the track [of] cognitive of events across time can be unreliable, a fleeting memory (p. 18).

In addition, Efklides (2011) indicated:

This conception of the SRL functioning at the Task × Person level presupposes a cognitive architecture in which there are conscious analytic processes and explicit knowledge as well as non-conscious automatic processes and implicit knowledge that have a direct effect on behavior (p. 13).

Boekaerts also assumed that automaticity can play a crucial role in the different pathways that students might activate: “Bargh’s (1990) *position is that goal activation can be automatic or deliberate and* Bargh and Barndollar (1996) *demonstrated that some goals may be activated or triggered directly by environmental cues, outside the awareness of the individual*” (Boekaerts and Niemivirta, 2000, p. 422). Pintrich (2000) specified: “*At some level, this process of activation of prior knowledge can and does happen automatically and without conscious thought*” (p. 457). Finally, Zimmerman and Moylan (2009) asserted:

In terms of their impact on forethought, process goals are designed to incorporate strategic planning-combining two key task analysis processes. With studying and/or practice, students will eventually use the strategy automatically. Automization occurs when a strategy can be executed without close metacognitive monitoring. At the point of automization, students can benefit from outcome feedback because it helps them to adapt their performance based on their own personal capabilities, such as when a basketball free throw shooter adjusts their throwing strategy based on the results of their last shot^3^. However, even experts will encounter subsequent difficulties after a strategy becomes automatic, and this will require them to shift their monitoring back from outcomes to processes (p. 307).

Thus, automaticity is an important aspect in the majority of the models. Here, there are three aspects for reflection. First, there are automatic actions that affect SRL; for example, Pintrich (2000) mentioned access to prior knowledge and Boekaerts (2011) discussed goal activation. Second, we can assume that even self-regulation, when it is understood to be the enactment of a number of learning strategies to reach students’ goals, can happen implicitly, as proposed by Winne (2011). This means that students can be so advanced in their use of SRL strategies that they do not need an explicit, conscious, purposive action to act strategically. Nevertheless, this takes practice. Third, some automatic reactions, particularly some emotions, and even some complex emotion-regulation strategies may not be positive for learning (Bargh and Williams, 2007). For example, Boekaerts (2011) mentions that the well-being pathways can be activated even when students are not aware. Therefore, assisting students to become aware of those negative automatic processes could have the potential to enhance self-regulation that is oriented toward learning.

#### Context

The SSRL model emphasizes not only the role of context, but also the ability of different external sources (group members, teachers, etc.) to promote individual self-regulation by exerting social influence (CrRL) or of groups of students to regulate jointly while they are collaborating (SSRL) (Järvelä and Hadwin, 2013). Zimmerman (2000) did not include context in his Cyclical Phases model, only a minor reference to the specific strategy “environmental structuring.” However, in his Triadic and Multi-level models, the influence of context and vicarious learning is key to the development of self-regulatory skills (Zimmerman, 2013). Boekaerts and Niemivirta (2000) posits that students’ interpretation of the context activates different goal pathways and that previous experiences affect the different roles that students adopt in their classrooms (e.g., joker, geek). For Winne (1996), Pintrich (2000), and Efklides (2011) models, context is: (1) important to adapt to the task demands, and (2) part of the loops of feedback as students receive information from the context and adapt their strategies accordingly. In sum, all of the models include context as a significant variable to SRL. Nevertheless, with the exception of Hadwin, Järvelä, and Miller’s work, not much research has been conducted by the others in exploring how significantly other contexts or the task context affect SRL.

## Discussion

The aim of this review was to compare educational psychology models of SRL. To achieve this goal, the included models have been presented and compared. Next, some final conclusions will be presented.

### Meta-Analytical Empirical Evidence for the Models

All of the models have empirical evidence that supports the validity to some of their main aspects. However, because the SRL models share a high number of processes, there is a significant overlap in the empirical evidence. For example, self-efficacy is a crucial variable for some SRL models (e.g., Pintrich, 2000; Zimmerman, 2000). Thus, if we consider van Dinther et al. (2010) review on factors affecting self-efficacy in higher education students, it has implications for all those models that emphasize self-efficacy as a crucial SRL process. For this reason, trying to disentangle each individual empirical contribution tailored to a specific SRL model and applying it to the other five models would be very complex. As a consequence, the analysis will focus on more transversal findings, which stem from the meta-analyses conducted in the SRL field.

Three meta-analyses have been conducted with the main aim to study SRL effects (Dignath and Büttner, 2008; Dignath et al., 2008; Sitzmann and Ely, 2011) and a fourth meta-analysis have explored *learning skills interventions* with direct implications for SRL research (Hattie et al., 1996). First, regarding Hattie et al. (1996), they did not explore differential effects of SRL theories or models. Despite this limitation, one interesting conclusion was that: “it is recommended that training (a) be in context, (b) use tasks within the same domain as the target content, (c) and promote a high degree of learner activity and metacognitive awareness” (Hattie et al., 1996, p. 131). Secondly, Dignath’s work explored the effects of SRL interventions in primary-school students (Dignath et al., 2008), while Dignath and Büttner (2008) added secondary-education students. As extracted from the latter, regarding primary school results, effects size on academic performance were higher if “the intervention was based on social-cognitive theories (*B* = 0.33) rather than on metacognitive theories (reference category)” plus “if interventions also included the instruction of metacognitive (*B* = 0.39) and motivational strategies (*B* = 0.36)” (p. 246). In the same study, effects sizes for secondary education results about the same variables were found to be higher:

“if the intervention was based on metacognitive theoretical background (reference category) rather than on social-cognitive (*B* = -1.41) or motivational theories (*B* = -0.97)” plus “if the intervention focused on metacognitive reflection (*B* = 0.82) or motivation strategies (*B* = 0.56) rather than on cognitive strategies (reference category), but higher for interventions promoting cognitive rather than metacognitive strategies (*B* = -0.64)” (p. 246).

The last meta-analysis, by Sitzmann and Ely (2011), focused on how adults regulate their learning in two settings: higher education and the workplace. These authors did not include the SRL theoretical background as a moderator, as Dignath and colleagues did. However, they extracted three key results that apply to the aim of this review. First, the authors found that the constructs that there were included in more SRL theories were the ones that had stronger effects on learning (p. 433). Second, the overlap of the empirical results indicates that significant relationships exists between the different models. Third, “most of the self-regulatory processes exhibited positive relationships with learning, goal level, persistence, effort, and self-efficacy having the strongest effects. Together these four constructs accounted for 17% of the variance in learning after controlling for cognitive ability and pre-training knowledge” (p. 438).

Three main conclusions can be extracted from these four meta-analyses. First, SRL is a powerful umbrella to anchor crucial variables that affect learning, offering, at the same time, a comprehensive framework that explains their interactions. Second, SRL interventions are successful ways to improve students’ learning, if properly designed. Third, SRL interventions have differential effects based on the students’ educational level.

Self-regulated learning interventions that are grounded in socio-cognitive theory (Bandura, 1986) have a higher impact when used at earlier educational stages (e.g., primary), and when a framework is provided for students and teachers (Dignath et al., 2008). It has been hypothesized that this probably happens because socio-cognitive models (e.g., Zimmerman, 2000) are more comprehensive and easier to understand (Dignath et al., 2008). In addition, these models contain motivational and emotional aspects, which are more salient for academic performance during primary education (Dignath et al., 2008). When it comes to more mature students (i.e., those in secondary-education), they benefit more from interventions including more metacognitive aspects (Dignath and Büttner, 2008). This is probably due to the increased performance of cognitively demanding tasks in which it is necessary to use more specific strategies (Dignath and Büttner, 2008). Therefore, it could be hypothesized that metacognitive models (e.g., Efklides, Winne, and Hadwin) would have a higher impact at this educational level. Finally, the results from higher education and workplace trainees (Sitzmann and Ely, 2011) show that the four biggest predictors that were found—goal level, persistence, effort, and self-efficacy—have a significant motivational value and are all comprehended in the socio-cognitive theory. These results align with those of Richardson et al. (2012), who found that (a) self-efficacy was the highest predictor, (b) goal-setting strategy boosts effort regulation, and (c) multifaceted interventions may be more effective (pp. 375–376); and with the results of Robbins et al. (2004), where the best predictors for GPA were academic self-efficacy and achievement motivation. Therefore, there seems to be a tendency for higher education students to have better results if the interventions are aiming at motivational and emotional aspects—i.e., self-efficacy and goal setting. It could, thus, be hypothesized that models with an emphasis on motivation and emotion (e.g., Boekaerts, Pintrich, and Zimmerman) might have a higher impact. Nevertheless, it is important to emphasize that the conclusions regarding higher education students are not build on meta-analyses that explicitly compared different SRL models (i.e., theoretical backgroud) interventions as are the ones for primary and secondary education.

Finally, there was another interesting finding from Dignath and colleagues meta-analyses. They found that SRL interventions, including group work, were detrimental for primary students but that they had positive impact in secondary education students (Dignath and Büttner, 2008). This finding implies that the implementation of SSRL model interventions should be carefully conducted in primary education to try to maximize positive effects. Additionally, SSRL interventions might be needed in secondary and higher education, where the amount of group work increases.

### Educational Implications

Four educational implications will be discussed. First, if we examined the psychological correlates (e.g., self-efficacy, effort regulation, procrastination) that influence academic performance (Richardson et al., 2012), the conclusion is that the vast majority of these correlates are included in the SRL models. Additionally, SRL interventions promote students’ learning (Dignath et al., 2008; Rosário et al., 2012). Therefore, a first implication is that teachers need to receive training on SRL theory and models to understand how they can maximize their students’ learning (Paris and Winograd, 1999; Moos and Ringdal, 2012; Dignath-van Ewijk et al., 2013). There are three ways of intervening. First, pre-service teachers should receive pedagogical training for their future adaptation to the workplace. There is a significant number of studies conducted with pre-service teachers (e.g., Kramarski and Michalsky, 2009; Michalsky and Schechter, 2013), however, there is need for longitudinal studies exploring the final outcomes of such training when they start working. Second, in-service teachers also need to receive training on SRL, as they most probably did not receive any during their pre-service preparation (Moos and Ringdal, 2012). Third, teachers should gain SRL expertize themselves as learners, as this will impact their knowledge and pedagogic skills (Moos and Ringdal, 2012).

A second implication relates to how to teach SRL at different educational levels. Different models work better at different educational levels (Dignath and Büttner, 2008). Furthermore, another review shows that teachers at different educational levels used different approaches to SRL (Moos and Ringdal, 2012), but not in the expected direction. These authors found that: (a) higher education teachers tend to focus on the course content, providing limited opportunities for scaffolding SRL; (b) secondary teachers offer more of those opportunities but do not formulate explicit instructions in terms of SRL; and (c) primary teachers implement more SRL practices. There is, therefore, a misalignment between what SRL research says about its implementation at different educational levels (Dignath and Büttner, 2008), and what teachers actually do in their classroom (Moos and Ringdal, 2012). This brings us back to the previous implication: more teacher training is then needed, however, it needs to be tailored so that the interventions take the differential effects of SRL models into account.

A third implication is related to creating environments that leads students’ actions toward learning. All of the models consider SRL as goal-driven, so students’ goals direct their final self-regulatory actions. However, as Boekaerts (2011) argues, students also activate goals not oriented to learning (well-being pathway) and, as a consequence, students might self-regulate toward avoidance goals (e.g., pretending they are sick to miss an exam) (Alonso-Tapia et al., 2014). There is a line of research that explores how teachers can create a classroom environment that is conducive toward learning goals (Meece et al., 2006; Alonso-Tapia and Fernandez, 2008). Educators need to maximize the learning classroom climate for SRL to promote learning.

Fourth, a SRL skill developmental approach is more beneficial for learning. We already know that SRL skills develop over time with practice, feedback, and observation (Zimmerman and Kitsantas, 2005). We also know that students experience a high cognitive load when performing novel tasks, as claimed by cognitive load theory (Sweller, 1994). If we consider what we know on how to design instructional environments to minimize the impact of cognitive load (Kirschner, 2002), then a SRL skill developmental approach should be chosen. Such an approach would consider the four stages for acquisition of SRL, formulated in Zimmerman’s Multi-Level model (Zimmerman and Kitsantas, 2005): observation, emulation, self-control (including automaticity), and self-regulation. This approach will maximize SRL skill development and has been proposed for self-assessment, which is a crucial process for SRL (Panadero et al., 2016).

### Future Research Lines

Four future lines will be discussed. First, a call for a connection between the empirical evidence on SRL and meta-analytic evidence of correlates of learning and academic performance should be issued (e.g., Hattie et al., 1996; Richardson et al., 2012). As already argued, SRL models are comprehensive models. Therefore, the validation of the models becomes complex, as it requires either (a) conducting one study with a very large number of variables, or (b) conducting a number of studies with a narrower approach. However, if future research combines conclusions from previous meta-analyses (e.g., Hattie, 2009) and SRL models validational studies, we could advance our understanding of SRL and test even more specific SRL models’ differential effects. This attempt to combine meta-analytic and primary research studies should lead to the construction of a meta-model of SRL, including all SRL areas and interconnecting the existing models. A preliminary attempt can be found in Sitzmann and Ely (2011), however, it needs to be developed further.

Second, more fine-grained studies should also be conducted to understand how the specifics of SRL work. Although SRL models provide a quite specific picture of their processes, there is still much needed to understand SRL mechanisms more precisely (e.g., how self-reflection works, interactions that leads to attributions). This could be achieved through solid experimental designs controlling for strange variables.

Third, longitudinal research on the development of SRL skills throughout the life span is needed, especially regarding how SRL applies to adults in their workplace (Sitzmann and Ely, 2011). There is a compelling amount of research on SRL development during formal education years (Paris and Newman, 1990; Ley and Young, 1998; Núñez et al., 2013). However, we need to further implement longitudinal studies that cover a significant amount of years, and emphasize the role of SRL in adult life. In terms of the latter, our call would be to first test if the available models are valid, rather than developing a new SRL model (Sitzmann and Ely, 2011). Additionally, more longitudinal research on SRL, which focuses on its development during more specific and shorter periods of time, is needed. For example, studies that focus on one specific crucial academic year (e.g., first year of university). The research on learning diaries (Schmitz et al., 2011) is a very promising stream of research that allows for extraction of information in a longitudinal manner.

Fourth, new insights into SRL processes will come from recent developments in SRL measurement (Panadero et al., 2015b). The introduction of computers in SRL research, not only to measure but also to scaffold SRL, is showing promising results. This will provide more tailored interventions and learning environments over the coming years, which should be integrated into the existent body of knowledge.

## Conclusion

Self-regulated learning is a broad field that provides an umbrella to understand variables that influence students’ learning. Over the last two decades, SRL has become one of the major areas of research in educational psychology, and the current advances in the field are a signal that its relevance will continue. One conclusion from this review is that the SRL models are beneficial for interventions under different circumstances and populations, an aspect that need to be further considered by researchers and practitioners. Additionally, SRL models address a variety of research areas (e.g., emotion regulation, collaborative learning) and, therefore, researchers can utilize those that better suit their research goals and focus. Having a repertoire of models is enriching because researchers and teachers can tailor their interventions more effectively. Finally, I would like to issue a call for a new generation of researchers to take the lead in developing new approaches, measures, and, of course, SRL models—or to continue validating the ones that already exist. These future advances should promote changes in our understanding of SRL and the means through which research is conducted.

## Author Contributions

EP has acted as only author and lead the writing and editorial process of this submission.

## Conflict of Interest Statement

The author declares that the research was conducted in the absence of any commercial or financial relationships that could be construed as a potential conflict of interest.

## Footnotes

1 A third SRL handbook is under preparation edited by Dale Schunk and Jeffrey Greene.

2 An early draft of the chapter was provided by the authors for this review. The estimated year of publication for the handbook is 2018.

3 This was edited by Zimmerman in a personal communication. The original quote was: “on their height.”
